# National Medical Expenditures Associated With Surgical Care Among Individuals in the United States

**DOI:** 10.1097/AS9.0000000000000634

**Published:** 2025-12-16

**Authors:** Cornelius B. Groenewald, Jennifer A. Rabbitts, Elizabeth De Souza, T. Anthony Anderson, Mark C. Bicket

**Affiliations:** From the * Department of Anesthesiology & Pain Medicine, Department of Anesthesiology, Perioperative, and Pain Medicine, Stanford University School of Medicine, Stanford, CA; † Department of Anesthesiology, University of Michigan, Ann Arbor, MI.

## INTRODUCTION

Approximately 1 in 9 individuals undergo a surgical procedure in the United States each year.^1^ Using 2005 data, Muñoz et al^2^ estimated that surgical care accounted for nearly one-third of all US healthcare expenditures and predicted that surgical expenditures would significantly increase to represent one-fourteenth of the overall US economy by 2025. Moreover, surgical care alone comprises approximately half of total Medicare spending, underscoring its outsized impact on public health budgets.^3^ The present study uses data from the Medical Expenditure Panel Survey (MEPS) to provide contemporary data on national medical expenditures associated with surgical care, identify primary drivers of expenditures, and determine whether surgical expenditures as a proportion of overall healthcare expenditures have increased or decreased in the United States. Understanding national surgical expenditure is essential for policymakers as they allocate investments in training, infrastructure, and workforce planning to meet future needs.

## METHODS

This cross-sectional study used data from the MEPS, which is a set of national surveys conducted annually by the Agency for Healthcare Research and Quality, most recently in 2022.^4^ The primary aim of MEPS is to provide comprehensive estimates of medical care use, including surgery, among the US population.^5^ MEPS classifies participants as having undergone a surgical procedure (binary: Yes/No) if they had at least 1 inpatient stay, hospital outpatient visit, office-based visit, or emergency department visit during the past 12 months, which included an “operation or surgical procedure, including any procedure that involves cutting into the skin, including stitching of cuts and wounds.”^6^ This definition of surgery addresses a wide range of conditions from common procedures to complex, life-saving interventions, and was specified by MEPS to provide a high-level overview of the total expenditures associated with surgical care. Our primary outcome was total medical expenditures, defined as the sum of payments from a comprehensive range of health services, including inpatient and outpatient care, office-based visits, emergency room services, prescribed medications, and other medical supplies and equipment for medical care across health service categories. We captured expenditures both for the total MEPS sample and further categorized by surgery and nonsurgery samples (Table 1). We also analyzed expenditures by service category (inpatient, outpatient, etc) and source of payment (private, Medicare, etc) (Table 1). Based on goodness-of-fit testing comparing models, we used multivariable gamma generalized linear models with a square root link function to estimate adjusted associations between surgery and expenditures, robustly controlling for age, sex, race and ethnicity, family income, US census region, and comorbid conditions. We estimated margins from fitted models to assess incremental expenditures. Therefore, reported expenditures use model-based marginal effects. National incremental expenditures for surgery were estimated by multiplying the model-adjusted marginal expenditure difference ($12,349) by the weighted number of individuals undergoing surgery (39.4 million). Analyses were conducted with Stata version 19.5 (StataCorp, College Station, TX). We adjusted for the complex probability survey design of MEPS. The Stanford University IRB exempted this study from review.

**TABLE 1.** Mean Per Person Medical Expenditures of Individuals who had Surgery During 2022 as Compared With Those who did not Have Surgery, Adjusted for Sociodemographic and Comorbid Conditions*

	Individuals who had Surgery (A)		Individuals who did not had Surgery (B)		Incremental Costs Associated With Surgery (A–B)		
	$	95% CI	$	95% CI	$	95% CI	*P*
Total	17,300	(15,386,19,214)	4951	(4580,5322)	12,349	(10,424,14,273)	<0.0001
Service category							
Inpatient	5676	(4816,6536)	534	(441,626)	5142	(4306,5978)	<0.0001
Outpatient	4800	(4120,5479)	1411	(1292,1530)	3388	(2737,4040)	<0.0001
Office based	3538	(3114,3962)	450	(381,520)	3087	(2659,3515)	<0.0001
Prescribed meds	2621	(1275,3967)	1616	(1352,1879)	1005	(−237,2248)	0.11
Emergency room	664	(480,848)	188	(162,214)	476	(306,645)	<0.0001
Other	1465	(1211,1719)	790	(704,877)	674	(414,934)	<0.0001
Source of payment							
Private	8829	(7834,9823)	2433	(2174,2692)	6395	(5458,7332)	<0.0001
Medicare	4736	(4053,5419)	759	(623,896)	3976	(3283,4670)	<0.0001
Out of pocket	2248	(1786,2709)	744	(673,816)	1503	(1060,1945)	<0.0001
Medicaid/CHIP	2138	(757,3518)	810	(635,986)	1327	(76,2577)	0.037
VA	327	(185,468)	72	(53,92)	254	(114,394)	0.0004
Tricare	206	(56,356)	45	(19,72)	160	(−2,324)	0.053812
Other combined	248	(126,370)	103	(79,127)	145	(23,267)	0.019

* Multivariable gamma generalized linear models with square root link function examining associations between medical expenditure and surgery controlled for age, sex, race and ethnicity, family income, US census region, as well as the leading cost-driving medical conditions among adults in the United States (hypertension, angina, coronary heart disease, myocardial infarction, other heart disease, stroke, emphysema, high cholesterol, cancer, asthma, attention deficit disorder, diabetes, mental health conditions, and trauma-related conditions). Other expenditures include “other medical supplies and equipment”.

CHIP indicates children's health insurance program; CI, confidence interval; VA, veterans administration.

Data source: Medical Expenditure Panel Survey 2022.

## RESULTS

The study sample included 17,664 participants representing 253 million US adults with a mean age (standard error) of 48.6 (0.24) years, 51% female, and 17.3% Hispanic, 61.1% non-Hispanic White, 12% non-Hispanic African American, 6.5% non-Hispanic Asian American, and 3.0 % non-Hispanic other race. Overall, 15.5% (95% confidence interval: 14.9–16.2%) of participants reported having surgery in 2022, representing 39.4 million individuals nationwide.

The annual medical expenditures (after adjusting for covariates) for a participant who had surgery in 2022 were $17,300 as compared with $4951 for a participant who did not have surgery, a greater than threefold increase after controlling for multiple covariates. Consequently, incremental expenditures associated with surgery were $12,349 (95% confidence interval: $10,424–$14,273, *P* < 0.0001). The primary drivers of expenditures were inpatient stays ($5142), followed by hospital outpatient visits ($3388), and office-based visits ($3087). Expenditures on surgery were primarily borne by private insurance ($6395) and Medicare ($3976), while (on average) surgical patients paid $1503 out of pocket. We estimated national medical expenditures among all adults in the United States at $1.76T, with the national expenditures associated with surgery at $489B.

## DISCUSSION

We estimated the incremental expenditures of surgery in adults at $12,439 per person, amounting to $489 billion being spent on surgical care nationally. To our knowledge, this is the first study to report overall surgical expenditures using MEPS, the primary source of national medical expenditures in the United States. Our finding that surgery represented about one-third of all medical expenditures in the United States ($489B vs $1.76T) is consistent with existing, which found that surgical care accounted for 29% of healthcare expenditures in 2005.^2^ Of note, our findings of total medical expenditures among adults ($1.76T) are consistent with previous findings from MEPS.^7^ However, our findings may underestimate total economic expenditures associated with surgery as productivity losses and lost wages were not included in the analysis.

Our data should be interpreted with the following limitations. Surgical procedures differ in their degree of invasiveness and urgency, but this level of detail is not captured by MEPS. This limitation is common among national datasets, which trade specificity for the ability to collect data on a wide range of variables across large, representative samples. Secondly, self-reported data may be affected by recall bias and uncertain reliability; however, MEPS follows up with participants’ healthcare providers, pharmacies, and insurance companies to verify data. Additionally, response bias may arise, for instance, if older individuals who are more likely to undergo surgery are more inclined to respond, or if healthier individuals who are less likely to have surgery are overrepresented.

In 2010, Muñoz et al^2^ forecast that surgical expenditures would represent one-fourteenth of the overall US spending with a significant impact on US standard of living. However, recent National Health Expenditure data revealed that health care spending as a proportion of overall US gross domestic product has remained stable,^8^ which means that surgical expenditures did not significantly increase as forecast by Muñoz’s study. Regardless, our findings could assist healthcare policymakers in designing effective health policies and in managing public programs like Medicare and Medicaid, by involving surgeons in decision-making. For example, perioperative surgical homes, which include surgeons and anesthesiologists, are associated with lower surgical costs.^9^ In addition, nearly 1 in 3 Americans still lack access to high-quality surgical care, requiring additional investments in surgical care for the nation.^10^ As the population ages, it is likely that surgical expenditures may grow in the future, necessitating investments in training, infrastructure, and workforce planning to meet future needs.
